# Down-regulation of MBD4 contributes to hypomethylation and overexpression of CD70 in CD4^+^ T cells in systemic lupus erythematosus

**DOI:** 10.1186/s13148-017-0405-8

**Published:** 2017-09-22

**Authors:** Wei Liao, Mengying Li, Haijing Wu, Sujie Jia, Nu Zhang, Yong Dai, Ming Zhao, Qianjin Lu

**Affiliations:** 1 Department of Dermatology, Hunan Key Laboratory of Medical Epigenomics, The Second Xiangya Hospital, Central South University, Changsha, Hunan 410011 China; 2 Department of Pharmaceutics, The Third Xiangya Hospital, Central South University, Changsha, Hunan 410013 China; 3 Department of Microbiology, Immunology and Molecular Genetics, School of Medicine, The University of Texas Health Science Center at San Antonio, San Antonio, TX 78229 USA; 4 Clinical Medical Research Center, The Second Clinical Medical College of Jinan University (Shenzhen People’s Hospital), Shenzhen, Guangdong 518020 China

**Keywords:** Systemic lupus erythematosus, MBD4, CD70, DNA methylation

## Abstract

**Background:**

Systemic lupus erythematosus (SLE) is an autoimmune disease that is characterized by lymphocytic infiltration and overproduction of autoantibodies, leading to significant morbidity and mortality. However, the pathogenesis of this disorder has not yet been completely elucidated. It has been reported that CD70, a B cell costimulatory molecule encoded by the gene *TNFSF7* (tumor necrosis factor ligand superfamily member 7), is overexpressed in CD4^+^ T cells from patients with SLE due to the demethylation of its promoter. We aimed to investigate the expression patterns of MBD4 (methyl-CpG binding domain protein 4) in CD4^+^ T cells and its contribution to the pathogenesis of SLE by increasing CD70 expression through epigenetic regulation.

**Results:**

Our results showed that the expression of MBD4 was significantly decreased in CD4^+^ T cells from SLE patients. We verified that transfection of MBD4 siRNA into healthy CD4^+^ T cells upregulated expression of CD70 and decreased the methylation level of the CD70 promoter. Overexpression of MBD4 inhibited CD70 expression and enhanced the DNA methylation level of CD70 in CD4^+^ T cells of SLE patients.

**Conclusion:**

Our results indicated that downregulation of MBD4 contributed to overexpression and hypomethylation of the CD70 gene in SLE CD4^+^ T cells. This modulation of MBD4 may provide a novel therapeutic approach for SLE.

## Background

Systemic lupus erythematosus (SLE) is a multi-systemic disease that causes significant morbidity and mortality [1]. SLE is characterized by uncontrolled T and B lymphocyte activation and over-production of autoantibodies [2]. Immune complexes are deposited in various tissues and organs, leading to serious inflammation and tissue damage [3, 4]. Multiple factors including susceptibility genes, environments, hormones, and infections are believed to contribute to the onset and progression of SLE, though the molecular mechanisms that initiate the autoimmune response are still unclear [5, 6]. Growing evidence has implicated epigenetic factors in the pathogenesis of SLE. Our previous studies have shown that DNA hypomethylation contributes to the auto-reactivity of T cells by increasing the expression of some autoimmune genes, such as CD70, CD11a, and CD40L in CD4^+^ T cells of SLE patients. However, the mechanisms that regulate the expression and DNA methylation of autoimmune-related genes in SLE still remain unclear [7–10].

Human MBD4 (methyl-CpG binding domain protein 4) contains a C-terminal monofunctional thymine-uracil DNA glycosylase and N-terminal methyl-CpG binding domain separated by a region of unknown function [11–13]. MBD4 is a multifunctional protein involved in several cellular processes, including apoptotic response to DNA damage [14], transcriptional repression, and chromosomal stability [15]. Altered expression of MBD4 has also been observed in several autoimmune disorders; however, the role of MBD4 in the pathogenesis of autoimmune diseases remains unclear [16–18]. Previous studies have shown that MBD4 could function as a transcriptional repressor [15, 19]. MBD4 and its paralogs MBD1, MBD2, and MeCP2 recognize methylated DNA using their MBD domain and then inhibit downstream gene expression via a transcriptional repression domain, which itself recruits co-repressors, such as DNMT1 [20]. CD70, a B cell costimulatory molecule encoded by the *TNFSF7* (tumor necrosis factor ligand superfamily member 7) gene [21], is typically expressed by activated CD4^+^ T and CD8^+^ T cells and early B cell progenitors. It has been shown that overexpression of CD70 in human CD4^+^ T cells stimulates IgG synthesis in B cells in vitro [22].

CD70 gene demethylation is concomitant with increased expression in human T cells treated with traditional DNA methylation inhibitors [22]. CD4^+^ T cells from SLE patients exhibit similar demethylation patterns [8], as do those from patients with sjögren’s syndrome [23], subacute cutaneous lupus [24], and rheumatoid arthritis [25]. In this study, we compared the expression level of MBD4 in CD4^+^ T cells between SLE patients and healthy subjects and investigated whether MBD4 was involved in regulating CD70 expression and methylation status. Our study indicated that MBD4 downregulation contributed to overexpression and DNA hypomethylation of the CD70 gene in SLE CD4^+^ T cells, suggesting that MBD4 plays an important role in regulating aberrant DNA methylation and autoimmune responses in SLE.

## Results

### Expression levels of MBD4 in CD4^+^ T cells of SLE patients

**Fig. 1:**
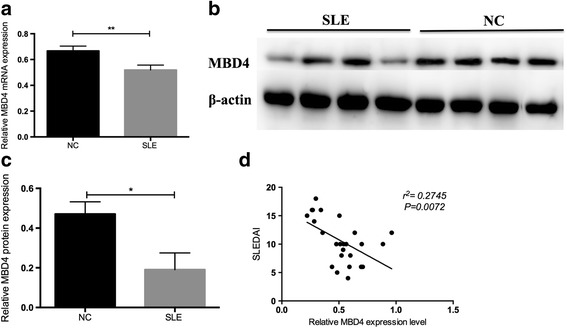
The expression level of MBD4 and its correlation with SLEDAI scores. **a** Comparison between the MBD4 mRNA expression levels in CD4^+^ T cells from SLE patients and healthy controls. **b**, **c** Comparison of MBD4 protein expression levels between the CD4^+^ T cells from SLE patients and healthy controls. Representative results of western blot are shown in (**b**), and quantitative analysis of the band intensities normalized to β-actin is shown in (**c**). **d** SLEDAI is short for Systemic Lupus Erythematosus Disease Activity Index. We can assess the severity of the disease according to the scores. MBD4 mRNA expression levels in the CD4^+^ T cells of SLE patients were negatively correlated with SLEDAI scores (*r* ^2^ = 0.2745, *P* < 0.01). Horizontal bars represent the mean ± SEM. **P < 0.01, *P < 0.05

To explore the role of MBD4 in SLE, we first measured the expression levels of MBD4 in CD4^+^ T cells from 25 SLE patients and 29 healthy subjects using RT-qPCR. The results showed that the MBD4 mRNA expression levels were significantly lower in SLE CD4^+^ T cells than those in healthy controls (Fig. 1a). The results of western blot also showed that the MBD4 protein levels were downregulated in the CD4^+^ T cells of SLE patients compared with healthy controls (Fig. 1b, c). In addition, we observed an obvious negative correlation between the MBD4 mRNA expression levels and the Systemic Lupus Erythematosus Disease Activity Index (SLEDAI) scores (Fig. 1d).

### Knockdown of MBD4 upregulates CD70 expression in normal CD4^+^ T cells

**Fig. 2:**
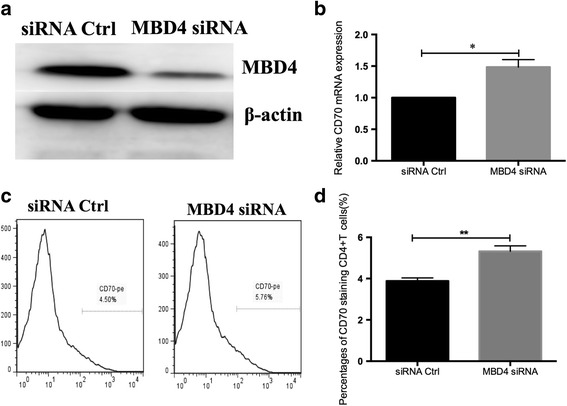
Knockdown of MBD4 upregulates CD70 expression in normal CD4^+^ T cells. **a** Western blot showing the decreased expression of MBD4 protein in CD4^+^ T cells 48 h post-transfection of MBD4 siRNA. **b** The mRNA level of CD70 was significantly upregulated in CD4^+^ T cells transfected with MBD4 siRNA compared with negative control. **c**, **d** Transfected cells were stained with anti-CD4-FITC antibody and anti-CD70-PE antibody and analyzed using flow cytometry (**c**). The percentages of CD70-staining CD4^+^ T cells are shown in (**d**). We first gated on CD4^+^ T cells and then defined CD70-positive T cells (**c**). All data represent the mean of three independent experiments per group (**P < 0.01, *P < 0.05)

To investigate whether MBD4 regulates CD70 gene expression in CD4^+^ T cells, MBD4 expression was inhibited using RNA interference (RNAi) in CD4^+^ T cells from healthy subjects. Transfected CD4^+^ T cells were harvested 48 h post-transfection. Western blots were then performed to determine the efficiency of gene silencing (Fig. 2a). Flow cytometric analysis confirmed that MBD4 knockdown caused upregulation of the CD70 level in normal CD4^+^ T cells (Fig. 2c, d). Moreover, the results of RT-qPCR also confirmed that knockdown of MBD4 upregulated the mRNA level of CD70 in normal CD4^+^ T cells (Fig. 2b).

### Upregulation of MBD4 represses CD70 expression in SLE CD4^+^ T cells

**Fig. 3:**
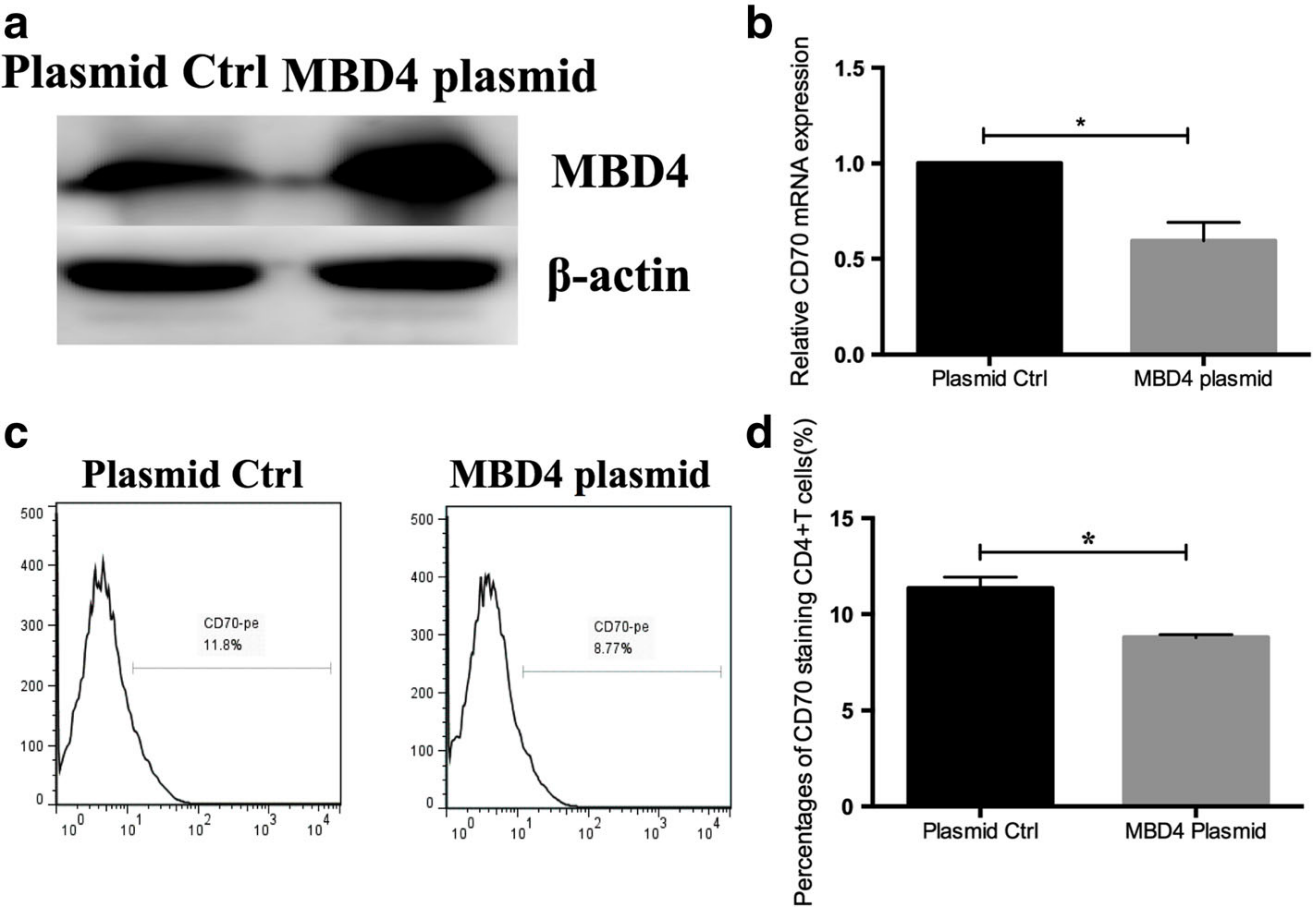
Upregulation of MBD4 represses CD70 expression in SLE CD4^+^ T cells. **a** The western blot results show the increased expression level of MBD4 protein in CD4^+^ T cells after 48 h post-transfection of MBD4 expression plasmid. **b** The mRNA level of CD70 was decreased in SLE CD4^+^ T cells transfected with the MBD4 plasmid compared with the negative control. **c**, **d** Transfected cells were stained with anti-CD4-FITC antibody and anti-CD70-PE antibody and analyzed with flow cytometry (**c**). The percentages of CD70-staining CD4^+^ T cells are shown in (**d**). All data represent the mean of three independent experiments per group (*P < 0.05)

To investigate the role of downregulation of MBD4 in CD4^+^ T cells of SLE patients, we increased MBD4 gene expression by transfecting the MBD4 expression plasmid pcDNA3.1-MBD4 into SLE CD4^+^ T cells. The western blot results confirmed that MBD4 expression was significantly increased in SLE CD4^+^ T cells transfected with the MBD4 expression plasmid compared with the negative control (Fig. 3a). Furthermore, the results of RT-qPCR and flow cytometry indicated that CD70 mRNA and protein expression levels were clearly downregulated in SLE CD4^+^ T cells with MBD4 overexpression compared with the negative control (Fig. 3b–d).

### MBD4 regulates the methylation status of CD70 gene promoter in CD4^+^ T cells

**Fig. 4:**
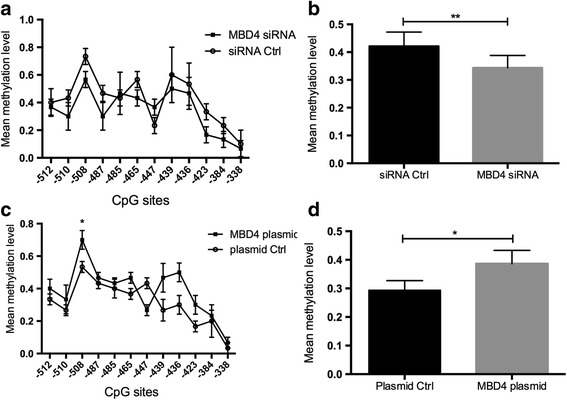
MBD4 regulates the methylation status of CD70 gene promoter in CD4^+^ T cells. **a** Comparison of the CD70 promoter methylation levels between normal CD4^+^ T cells transfected with MBD4 siRNA and negative control. Ten clones from each amplified fragment were sequenced. The methylation status of each CG pair (*X*-axis) within the region from − 581 to −288 bp upstream of the CD70 gene was assessed and indicated as the mean methylation status (*Y*-axis). **b** The average methylation level of 10 CG pairs (− 512, − 510, − 508, − 487, − 465, − 439, − 436, − 423, − 384, and − 338 bp) was significantly downregulated when MBD4 expression was inhibited in normal CD4^+^ T cells. **c** Comparison of CD70 promoter methylation levels between SLE CD4^+^ T cells transfected with MBD4 expression plasmid and negative control. **d** The mean methylation level of the region from − 581 to − 288 bp excluding one CG pair located in − 447 bp, was upregulated in SLE CD4^+^ T cells with MBD4 overexpression compared with negative controls. All data represent the mean of three independent experiments per group (**P < 0.01, *P < 0.05)

Previous studies have reported that the methylation status between − 581 and − 288 bp upstream of transcription starting site of CD70 gene influenced CD70 transcription, which was hypomethylated in CD4^+^ T cells from SLE [8]. To investigate whether MBD4 is involved in regulating the DNA methylation status of the CD70 promoter in CD4^+^ T cells, we first inhibited MBD4 expression and detected the DNA methylation level of the CD70 promoter in normal CD4^+^ T cells. The bisulfite sequencing results showed that the mean methylation level of the 294 bp sequence (from − 581 to − 288 bp) upstream of CD70 gene that included 10 CG pairs (located at − 512, − 510, − 508, − 487, − 465, − 439, − 436, − 423, − 384, and − 338 bp) was downregulated significantly when MBD4 expression was inhibited in normal CD4^+^ T cells (Fig. 4a, b). By contrast, when MBD4 expression was overexpressed in SLE CD4^+^ T cells, we detected the DNA methylation status in CD70 gene promoter. This result showed that the mean methylation level of these 294 bp sequences, excluding one CG pair located at − 447 bp, was increased significantly in SLE CD4^+^ T cells with MBD4 overexpression compared with negative controls (Fig. 4c, d).

### MBD4 regulates the transcription activity of CD70 promoter by altering its promoter methylation status

**Fig. 5:**
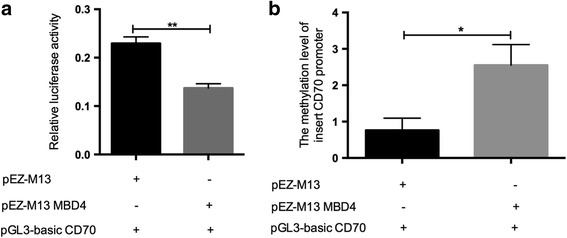
MBD4 regulates the CD70 promoter activity by changing promoter methylation status. **a** Relative firefly luciferase activity in HEK293T cells co-transfected with an empty vector (pEZ-M13) or an MBD4-expressing vector (pEZ-M13 MBD4), together with luciferase reporter constructs containing the CD70 gene promoter. The luciferase activity was decreased in HEK293 T cells transfected with MBD4 expression vector compared with empty control vector, suggesting MBD4 represses the activity of CD70 promoter. **b** The result of R-MeDIP-qPCR showed that the methylation level of the inserted CD70 promoter in luciferase reporter vector was increased in HEK293T cells transfected with MBD4 expression vector compared with empty control vector. Three independent experiments were performed (**P < 0.01, *P < 0.05)

To confirm that CD70 is a target gene of MBD4, we constructed a firefly luciferase reporter plasmid that contained a segment of the CD70 promoter. HEK293T cells were co-transfected with the firefly luciferase reporter vector and MBD4 plasmid or a negative control, and the luciferase activity was measured 48 h later. The MBD4 overexpression significantly reduced luciferase activity of CD70 promoter (*P* < 0.01) (Fig. 5a). Taken together, these data suggested that MBD4 regulated CD70 gene transcription. To further verify whether the methylation level of the CD70 promoter is altered by MBD4, we used a proven reporter-based MeDIP (R-MeDIP) assay. After MeDIP, quantitative PCR was performed with specific primers to ensure that only reporter-promoter-specific DNA, but not genomic DNA, was amplified. As expected, the CD70 reporter-promoter was precipitated from transfected cell lysates with a 5-methylcytosine antibody. The results of quantitative RCP showed that 5-methylcytosine-mediated immunoprecipitation of CD70 reporter-promoters was significantly increased when the MBD4 plasmid was transfected (*P* < 0.05) (Fig. 5b). This experiment provides further evidence that the MBD4 affects CD70 gene transcription through regulating the methylation status of the CD70 promoter.

## Discussion

First, in addition to DNA methyltransferases (DNMTs), methyl-CpG-binding domain proteins (MBDs) have been shown to regulate gene expression by modulating DNA methylation of genes [26]. Second, our previous study showed that MBD4 and DNMT1 expression was decreased in CD4^+^ T cells from SLE patients compared with normal controls and was correlated with the global DNA methylation levels in CD4^+^ T cells [27]. So, we hypothesized that MBD4 might regulate the DNA methylation level of genes, similar to DNMT1. In this study, we chose to study the regulation of MBD4 on DNA methylation of CD70 promoter, because our previous studies have confirmed the hypomethylation of CD70 promoter region in SLE CD4^+^ T cells  [20, 28].

In this study, we confirmed that the MBD4 protein and mRNA levels were downregulated and negatively correlated with SLEDAI. Abnormal expression of MBD4 has been reported in several other diseases, for example, SLE, systemic sclerosis (SSC), primary immune thrombocytopenia (ITP), and vitiligo. MBD4 expression is decreased in systemic autoimmune diseases, such as SLE and SSC [27], but is increased in organic autoimmune diseases, such as ITP and vitiligo [17, 29]. Additionally, MBD4 expression is correlated with the global DNA methylation level in SSC [27]. One reason for the difference of MBD4 expression among these autoimmune diseases may be that the SLE is a systemic disease, not an organ-specific autoimmune disease, such as vitiligo [30]. Therefore, the abnormal disease-specific expression level of MBD4 in SLE CD4^+^ T cells may have unique effects on immune activation, which causes deterioration into a systemic disease in SLE rather than an organ-specific autoimmune disease. Our previous studies on some organ-specific autoimmune diseases, such as latent autoimmune diabetes in adults (LADA) and vitiligo, also suggest that a massive gap exists between organ-specific diseases and systemic diseases [17, 31]. Another explanation for the abnormal gene expression level in SLE CD4^+^ T cells is that the cell populations (PBMCs and CD4^+^ T cells) that we investigated differed compared with others [32]. Unlike a tricky mixture of PBMCs, CD4^+^ T cells were not affected by other cells, such as monocytes and macrophages.

Our results demonstrated that transfecting the MBD4 plasmid into active T cells from SLE patients inhibited the expression of CD70, which was identified by flow cytometry and western blot, suggesting that MBD4 represses the expression of CD70 in T cells. Furthermore, the luciferase reporter assay and R-MeDIP experiment confirmed that CD70 promoter activity was regulated by MBD4 through modulating the DNA methylation status of CD70 promoter. Several groups have shown that the CD70 gene is overexpressed and hypomethylated in SLE CD4^+^ T cells. Zhao et al. proved that the decreased DNMT1 promoted CD70 overexpression and DNA hypomethylation [28]. Shi et al. showed a significant correlation between the MBDs and DNMTs that indicated that they jointly regulated DNA methylation in SLE [20]. It has been reported that MBD4 cooperates with DNMT1 to mediate methyl-DNA repression and protects mammalian cells from oxidative stress [26]. These results further indicated that MBD4 might play a regulatory role with DNMT1. Surprisingly, MBD2 was reported to have the functions of both a transcriptional repressor and DNA demethylase [16]. MBD4 remains more controversial, though some studies also showed that MBD4 was associated with DNA demethylation. For example, Rai et al. previously reported that MBD4 mediates DNA demethylation in zebrafish embryos, by initiating base excision repair of G-T mispairs as well as by directing the actions of activation-induced deaminase (AID) and growth arrest and DNA damage-inducible protein 45 alpha (Gadd45a) [33]. However, some investigators hold opposite opinions. As reported by Detich et al., there is no evidence for AID-MBD4-mediated DNA demethylation in zebrafish embryos [34], which is in line with our results in humans. Remarkably, it is important to note that some reports failed to observe a defect in DNA methylation in MBD4 knockout mice [35]. Together, these current biological studies have not conclusively demonstrated a role for MBD4 in active DNA demethylation. In this study, we showed that MBD4 had an additional role in transcriptional repression through methyl-CpG, which is supported by the study performed by Kondo et al., suggesting that MBD4 is an essential component of epigenetic silencing [15].

## Conclusions

Our results demonstrated that MBD4 was downregulated, which lead to the overexpression and promoter hypomethylation of CD70 in SLE CD4^+^ T cells. This study preliminarily revealed the role and mechanism of MBD4 in the pathogenesis of SLE; however, further study is required to investigate the mechanism by which MBD4 regulates DNA methylation status of genes in CD4^+^ T cells of SLE.

## Methods

### Subjects

All SLE subjects (*n* = 25, mean age 35.52 ± 2.70 years) who fulfilled at least four of the SLE classification criteria of the American College of Rheumatology were recruited from outpatient clinics in the Second Xiangya Hospital, Central South University [36]. Disease activity was assessed by the SLEDAI at the time of blood collection [37]. Patient demographics, treatment regimens, and clinical data are shown in Tables 1 and 2. A total of 29 healthy controls (*n* = 29, mean age 38.52 ± 4.78 years) were recruited from the medical staff at the Second Xiangya Hospital and Changsha Blood Center. Patients and controls were matched for age and sex in all experiments. The human sample study was under a protocol approved by the Ethics Committee of Second Xiangya Hospital, Central South University, with an informed written consent.

**Table 1:** Patient demographics and medications

Patients	Gender	Age (years)	SLEDAI	Treatment regimen
1	F	25	9	None
2	F	45	10	Pred .20 mg/d HCQ.200 mg/d
3	F	22	10	None
4	F	59	12	Pred .15 mg/d HCQ.200 mg/d TG 30 mg/day
5	F	35	12	Pred .20 mg/d HCQ.200 mg/d
6	F	47	16	Pred .20 mg/d HCQ.200 mg/d TG 30 mg/day
7	F	41	4	Pred .10 mg/d HCQ.200 mg/d
8	M	47	16	Pred .10 mg/d TG 30 mg/day
9	F	23	15	Pred .15 mg/d HCQ.200 mg/d
10	F	47	14	None
11	F	47	6	Pred .15 mg/d HCQ.200 mg/d
12	F	22	15	Pred .40 mg/d HCQ.200 mg/d CTX. 800 mg/once
13	F	38	8	Pred .15 mg/d HCQ.200 mg/d
14	F	17	16	Pred .30 mg/d
15	F	49	5	Pred .7.5 mg/d HCQ.200 mg/d
16	F	19	12	Pred.17.5 mg/d HCQ.200 mg/d
17	F	40	6	Pred .15 mg/d HCQ.200 mg/d
18	M	46	6	Pred .10 mg/d
19	F	17	10	Pred .30 mg/d HCQ.200 mg/d
20	F	54	8	Pred .20 mg/d TG 30 mg/day
21	F	22	18	Pred .40 mg/d HCQ.200 mg/d
				CTX. 800 mg/once
22	F	25	10	Pred .30 mg/d HCQ.200 mg/d
23	F	54	10	Pred .12.5 mg/d HCQ.200 mg/d
24	F	22	6	Pred .5 mg/d HCQ.200 mg/d
25	F	25	10	Pred .15 mg/d HCQ.200 mg

*SLEDAI* Systemic Lupus Erythematosus Disease Activity Index, *F* female, *M* male, *Pred* prednisone, *HCQ* hydroxychloroquine, *CTX* cyclophosphamide, *TG* tripterygium glycosides, *none* no treatment with drugs

**Table 2:** Patients’ clinical data

Patients	Skin lesions	Joint injury	Hematologic abnormalities	Kidney damage	dsDNA	ANA
1	P	N	P	N	P	1:40
2	N	N	N	N	NR	NR
3	N	N	N	P	N	1:20
4	N	N	N	P	N	1:40
5	P	P	N	P	N	1:40
6	P	N	N	P	NR	NR
7	N	N	N	N	N	1:80
8	P	N	N	P	P	1:80
9	N	P	P	P	P	1:160
10	P	P	P	P	N	1:40
11	N	N	N	P	N	1:20
12	N	P	P	P	P	1:80
13	P	N	N	N	P	1:80
14	P	N	N	P	N	1:40
15	N	N	P	N	N	1:40
16	P	P	N	P	P	1:40
17	P	N	P	N	N	1:40
18	P	N	N	N	N	1:20
19	P	P	P	N	P	1:160
20	P	N	N	P	N	1:80
21	P	N	N	P	N	1:40
22	P	N	N	N	N	1:320
23	P	P	N	N	N	1:40
24	N	N	P	P	N	(−)
25	P	N	N	P	P	1:160

*N* negative, *P* positive, *NA* not recorded, *dsDNA* double-stranded DNA, *ANA* antinuclear antibody

### Cell isolation, cultures, and transfection

A total of 50 mL of venous peripheral blood was withdrawn from each patient and control subject and preserved with heparin. Peripheral blood mononuclear cells (PBMCs) were separated by density gradient centrifugation (GE Healthcare, Switzerland). CD4^+^ T cells were isolated with positive selection using Miltenyi beads according to the manufacturer’s instructions (Miltenyi, Germany) and cultured in human T cell culture medium (Gibco, California, USA) supplemented with 10% fetal bovine serum (FBS). Purified normal CD4^+^ T cells were transfected with plasmid or siRNA (Invitrogen, USA) using the Human T Nucleofector and Amaxa Nucleofector (Lonza, Switzerland). After 6 h of transfection, the medium was refreshed and the cells were seeded in anti-CD3 antibody (1 μg/ml) pre-coated 6-well plates in the presence of anti-CD28 antibody (1 μg/ml) and incubated for another 42 h. The cells were harvested for subsequent analysis. The sequence of MBD4 siRNA used were as follows: 5′-CAACGAGAAGUAUGAGGAUACCUUU-3′ (forward) and 5′-AAAGGUAUCCUCAUACUUCUCGUUG-3′ (reverse). The MBD4 siRNA control was purchased from Invitrogen (USA). The MBD4 plasmid was purchased from the corporation (Vigene Biosciences).

### RNA isolation and quantitative PCR (RT-qPCR)

Total RNA from CD4^+^ T cells was extracted using Trizol reagent (Invitrogen, USA). cDNA synthesis was performed with the PrimeScript® RT reagent kit with gDNA Eraser (TaKaRa Biotech Co., China) using 1 μg of total RNA according to the manufacturer’s instructions. The reaction mixture contained 2 μl of cDNA, 10 μl of SYBR Premix Ex TaqTM (TaKaRa Biotech Co., China), and 400 nM sense and antisense primers to a final volume of 20 μl. Transcripts were measured using a Roche-LightCycler96 Real-Time PCR System (Basel, Switzerland). The value of each cDNA was calculated using the 2^−ΔCt^ (− ΔCt = Ct_MBD4_ −  Ct_GAPDH_.) method and normalized to GAPDH. Primers used were as follows: GAPDH, 5′- ATGGGGAAGGTGAAGGTCG-3′ (forward) and 5′-GGGGTCATTGATGGCAACAATA-3′ (reverse); MBD4, 5′- TCTAGTGAGCGCCTAGTCCCAG-3′ (forward) and 5′-TTCCAATTCCATAGCAACATCTTCT-3′ (reverse); CD70, 5′- TGCTTTGGTCCCATTGGTCG-3′ (forward) and 5′-TCCTGCTGAGGTCCTGTGTGATTC -3′(reverse).

### Western blot

CD4^+^ T cells were lysed in protein lysis buffer and quantified by the Bradford assay (HyClone-Pierce, USA) followed by 8% vertical dodecyl sulfate-polyacrylamide gel electrophoresis and transferred to PVDF membranes (Millipore, USA). The membrane was blocked with 5% non-fat dry milk in Tris-buffered saline containing 0.1% Tween-20 (TBST) buffer and then incubated with primary antibodies including rabbit anti-human MBD4 (BioVison, USA) or mouse anti-human β-actin (ABclonal, USA), followed by HRP-goat anti-rabbit IgG antibody (ABclonal, USA) or HRP-rabbit anti-mouse IgG antibody (Santa Cruz, USA). Proteins were detected with an ECL Western blot detection kit (Thermo Scientific, USA). Quantification of MBD4 was normalized to β-actin by densitometry.

### Flow cytometry

To examine the expression of surface markers, cells were incubated with FcR blocking reagent (Miltenyi, Germany) for 10 min followed by primary antibodies on ice in the dark for 30 min. The antibodies used for surface marker analysis included anti-human CD4-FITC, CD70-PE (BD Pharmingen, USA); Data were acquired by flow cytometry (BD, Canto II, USA) and analyzed using FlowJo (Tree Star, USA).

### Plasmid construction and luciferase activity assay

The sequences of CD70 gene promoter spanning from − 628 to − 1 bp upstream of the transcription start site was inserted into pGL3-REPORT luciferase expression reporter vector (Genscript) using Kpn I and Hind III. The inserts were confirmed by DNA sequencing. HEK293T cells were cultured in DMEM with 10% FBS. Cells were plated in a 24-well plate. After overnight incubation, cells were co-transfected with 0.5 μg of firefly luciferase reporter vector and 1 μg of MBD4 plasmid (pEZ-M13 MBD4) or negative control (pEZ-M13) by Lipofectamine 2000 (Invitrogen). Every sample was co-transfected with 0.1 μg of pRL-TK plasmid expressing Renilla luciferase to monitor the transfection efficiency (Promega). After 48 h, cells were collected, washed twice, and suspended in 100 μl of passive lysis buffer (Promega). Firefly luciferase activity was measured using the Dual-Luciferase Reporter Assay System (Promega) with a GloMax 20/20 luminometer (Promega). Relative luciferase activity was normalized to renilla luciferase activity for each transfected well.

### Genomic DNA extraction and bisulphite sequencing

Genomic DNA was extracted from CD4^+^ T cells (QIANGEN DNA Extraction kit; QIAGEN, Germany). Two hundred to 500 ng of purified DNA was treated with sodium bisulfite, and bisulphite conversion was then performed (EpiTect Bisulphite kit; Qiagen) and a long fragment from upstream of CD70 gene (from − 581 to − 288 bp) was amplified by using two rounds of nested PCR with the primers. The sequence of these primers were described as following [23]: round 1: CD70, 5′-GGTGAATTCTTTAAGGTTAGGAGTTTAAGTTTAGTT-3′ (forward) and 5′-CAATCTAGAACTACACATTTATTAAAAATTAAATTA-3′ (reverse); round 2: CD70, 5′-GTTGAATTCGGTTAATATGGTGAAATTTTATTTTTAT-3′ (forward) and 5′-CACTCTAGATACAACAAACATCCAAAAATTAAAAATA-3′(reverse). Amplification products were then cloned into pGEM T vectors (Promega, Madison, WI, USA), and ten random clones were sequenced [38].

### Luciferase reporter methylated DNA immunoprecipitation (R-MeDIP)-qPCR

pGL3-REPORT luciferase expression plasmids containing the CD70 promoters and MBD4 plasmid were co-transfected into HEK293T cells. Forty-eight hours after transfection, DNA was extracted as described above. MeDIP analysis was performed according to the manufacturer’s instructions provided in the MeDIP assay kit (Active Motif, California, USA). Precipitated DNA was amplified with quantitative PCR using forward primers flanking the pGL3-basic vector sequence and the reverse primers flanking the inserted CD70 promoter sequence. CD70: 5′-CAAGTGCAGGTGCCAGAACA-3 (forward), 5′-GCCAACATGGTGAAACCCC-3′ (reverse).

### Statistical analysis

All statistical analyses were performed by SPSS software (version 17.0; Chicago, IL, USA). Results were expressed as mean ± SEM. Comparisons were compared using the Student’s *t* test. Correlations were determined using Pearson’ s correlation coefficient. *P* < 0.05 was considered significant.

## Abbreviation

ACR: American College of Rheumatology
AID: Activation-induced deaminase
ANA: Antinuclear antibody
CTX: Cyclophosphamide
DNMT: DNA methyltransferase
dsDNA: Double-stranded DNA
F: Female
FBS: Fetal bovine serum
Gadd45a: Grow arrest and DNA damage-inducible protein 45 alpha
HCQ: Hydroxychloroquine
ITP: Primary immune thrombocytopenia
M: Male
MBD: Methyl binding domain protein
MeDIP: Methylated DNA Immunoprecipitation
PBMC: Peripheral blood mononuclear cell
Pred: Prednisone
SLE: Systemic lupus erythematosus
SLEDAI: SLE Disease Activity Index
SSC: Systemic sclerosis
TG: Tripterygium
TNFSF: Tumor necrosis factor ligand superfamily
